# Immunogenic potential and neutralizing ability of a heterologous version of the most abundant three-finger toxin from the coral snake *Micrurus mipartitus*

**DOI:** 10.1590/1678-9199-JVATITD-2023-0074

**Published:** 2024-11-25

**Authors:** Luz Elena Romero Giraldo, Sergio Pulido, Mario Andrés Berrío, María Fernanda Flórez, Paola Rey-Suárez, Vitelbina Núñez-Rangel, Mónica Saldarriaga Córdoba, Jaime Andrés Pereañez

**Affiliations:** 1 Research Group in Toxinology, Pharmaceutical, and Food Alternatives, University of Antioquia, Medellín, Colombia.; 2 LifeFactors Free Zone SAS, Rionegro, Antioquia, Colombia.; 3 Tropical Disease Study and Control Program - PECET, University of Antioquia, Medellín, Colombia.; 4 Center for Research in Natural Resources and Sustainability, Bernardo O'Higgins University, Santiago, Chile.; 5 Microbiology School, University of Antioquia, Medellín, Colombia.

**Keywords:** Recombinant protein, Antibodies, Micrurus mipartitus, Coral snake antivenoms, Three-finger toxin, Mipartoxin-1

## Abstract

**Background:** *Micrurus mipartitus* is a coral snake of public health concern in Colombia. Its venom is mainly composed of three-finger toxins (3FTxs), Mipartoxin-1 being the most abundant protein partially responsible for its lethal effect. In this work, we present the production of Mipartoxin-1 in a recombinant form and evaluate its immunogenic potential. **Methods:** A genetic construct HisrMipartoxin-1 was cloned into the pET28a vector and heterologous expression was obtained in *E. coli* BL21 (DE3). The recombinant HisrMipartoxin-1 protein was extracted from inclusion bodies, refolded *in vitro*, and isolated by affinity and RP-HPLC chromatography. The lethal effect of HisrMipartoxin-1 was tested, and antibodies against HisrMipartoxin-1 were produced by immunization in rabbits. The antibody titers were monitored by an ELISA test. The neutralizing ability of the antibodies, against the lethal effect of native toxins and *M. mipartitus* venom, was also assessed. **Results:** HisrMipartoxin-1 was detected on SDS-PAGE, with a molecular mass of around 11 kDa. The retention time was 16.0 minutes. HisrMipartoxin-1 did not exhibit lethality in mice; however, antibodies against HisrMipartoxin-1 recognized the native toxin, the whole venom of *M. mipartitus*, and a 3FTx from another species within the *Micrurus* genus. Furthermore, antibodies against HisrMipartoxin-1 completely neutralized the lethal effect of native Mipartoxin-1 in mice but not *M. mipartitus* whole venom. **Conclusion:** These findings indicate that HisrMipartoxin-1 might be used as an immunogen to develop anticoral antivenoms or complement them. This work is the first report of the heterologous expression of 3FTx from *M. mipartitus*.

## Background

According to the World Health Organization (WHO), snakebite envenoming is a neglected disease and a severe public health problem that affects tropical and subtropical regions globally [1]. Millions of snakebite envenoming cases have been estimated worldwide, about 1.8-2.7 million people per year [2], with an estimated 81,000 to 138,000 deaths annually [1]. Of these, 137,000-150,000 envenomings and 3,400-5,000 deaths occur in Latin America and the Caribbean [2]. The Instituto Nacional de Salud (INS) of Colombia reported 5573 cases of snakebites in the year 2022, 1.3% being inflicted by coral snakes [3]. 

Coral snakes of the genus *Micrurus* (Wagler, 1824) are the representatives of the Elapidae family across the Americas, from the southern United States to Argentina [4]. Coral snakes have proteroglyphous fangs that allow them to inject venom and induce an envenomation that is characterized by neurotoxic effects such as bilateral ptosis and progressive respiratory paralysis [5]. According to Lomonte et al. [6] *Micrurus* venoms are predominantly composed of three-finger toxins (3FTxs) and Phospholipases A_2_ (PLA_2_s), the 3FTxs being the predominant proteins in *M. mipartitus* venom (~60% of total proteins) [7]. This species is widely distributed in Colombia and is commonly named ‘redtail coral snake’ or ‘rabo de ají’ [7]. Rey-Suárez et al. [8] identified Mipartoxin-1 (UniProt: B3EWF8) as the most abundant 3FTx from *M. mipartitus* venom (28%) and presents four isoforms [9]. Mipartoxin-1 is a short-chain (or “type-I”) α-neurotoxin of molecular mass 7030 Da and has a highly lethal activity in mice (LD50: 0.06 µg/g) [8]. Moreover, Mipartoxin-1 plays an important role in venom toxicity, contributing significantly to neurotoxic manifestations by causing neuromuscular blockade of post-synaptic nicotinic receptors, as was evidenced in both avian and mammalian preparations [8]. 

The standard treatment of coral snakebite is the administration of antivenom [10-14]. In Colombia, the National Institute of Health (INS) produces the Polyvalent Anticoral Antivenom, which is composed of equine immunoglobulins that neutralize the venom of *M. dumerilii*, *M. mipartitus*, *M. isozonus*, *M. surinamensis* and *M. lemniscatus*, *M. spixii* and *M. medemi* [15]. However, it is known that *M. mipartitus* and *M. dumerilii*, whose compositional dichotomy of their venoms differs between 3FTXs and PLA_2_, respectively, are the cause of most envenomings by coral snakes in Colombia [5, 7]. For this reason, it has been shown that, in some cases, the lethality of the venom has not been neutralized [16, 17] and that the neutralizing ability of antivenoms depends on the toxins they are directed towards.

The production of anticoral antivenoms is limited by the scarcity of *Micrurus* venoms. Their manufacture requires considerable quantities of the available venom for immunization procedures, but *Micrurus* species have small venom glands, producing low quantities of venom [18]. In theory, this difficulty could be resolved using many specimens. Still, *Micrurus* spp. have low survival in captivity due to low tolerance to changes in habitat and dietary preferences, which can lead to health problems [19]. Moreover, their terrestrial and semi-fossorial habits and their relatively small size make it difficult to find them in the field [20, 21].

The expression of key toxins in heterologous organisms of easy manipulation, inexpensive, and rapid growth as *Escherichia coli* [22], have contributed to overcoming the scarcity of antigens while minimizing dependence on wild or captive individuals used for obtaining the quantities of venom needed to produce anticoral antivenoms. Thus, some of *Micrurus* venom proteins have been expressed in recombinant form and used as immunogens [23]. Clement et al. [24] expressed the neurotoxin Mlat1 from the coral snake *Micrurus laticorallis* in two different *E. coli* strains using the expression vector pQE30, and the recombinant Mlat1-produced rabbit polyclonal antibodies recognized native Mlat1. Guerrero-Garzón et al. [25] obtained four transcript sequences (MlatA1, B.D, B.E, D.H) from the venom glands of *M. diastema*, *M. laticollaris*, *M. browni*, and *M. tener*, which encoding type-I α-neurotoxins. Toxin D.H was identified in *M. diastema* venom and expressed in Origami Gold DE3 *E. coli* strain using pQE30. In addition, an anti-rD.H serum neutralized the neurotoxic effects of *M. diastema* native α-neurotoxins. Likewise, de la Rosa et al. [26] obtained ScNtx α-neurotoxins from the twelve most toxic short-chain α-neurotoxins sequences of elapid venom from *Acanthophis, Oxyuranus, Walterinnesia, Naja, Dendroaspis,* and *Micrurus* genera. These authors expressed a consensus sequence of ScNtx α-neurotoxins in *E. coli* Origami using the same pQE30 plasmid. The antibodies against ScNtx recognized short-chain α-neurotoxins of elapid venoms. Also, Liu et al. [27] obtained three recombinant 3FTXs proteins from three Asian cobra species (*Naja kaouthia, Naja atra, Naja Siamensis*) using the pET-9a vector and an *E. coli* expression system. Immunization with each recombinant and a mixture of these (rsNTX, rLNTX, and rCTXA3) induced an immunological response to the native 3FTXs. Further, Ramos et al. [28] expressed recombinant multiepitope proteins in *E. coli* cells using the pAE vector. These authors developed an anti-elapid serum produced by a heterologous multiepitope DNA of most *M. coralinus* toxins. More recently, Romero-Giraldo et al. [29] expressed the most abundant PLA_2_ from *M. dumerilii* in *E. coli* using the pET28a vector. The recombinant His-rMdumPLA_2_ was biologically active, and anti-His-rMdumPLA_2_ antibodies recognized its native homologous and the complete venom of *M. dumerilii*. Since Mipartoxin-1 is the most abundant toxin in *M. mipartitus* venom and one of the main causes of envenomings by this species, the main goal of this research was to produce the heterologous expression of this 3FTx and evaluate its immunogenic potential. 

## Methods

### Bacterial strains, plasmids, and enzymes

*Escherichia coli* DH5α (Invitrogen, Waltham, MA, USA) and BL21(DE3) (Stratagene, San Diego, CA, USA) strains were used for cloning and protein expression, respectively. Plasmid pET28a (Novogene, Cambridge, UK) was used as an episomal vector to deliver genetic construct to the *E. coli* BL21 (DE3) strain. New England Biolabs (NEB) (Ipswich, MA, USA) NcoI, NotI, XhoI, EcoRV restriction enzymes, and T4 DNA ligase were used for the cloning process. 

### Plasmid construction 

**Figure 1.**
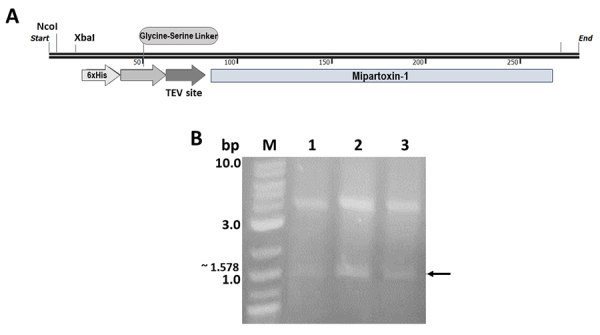
HisrMipartoxin-1 and digestion into pET28a. **(A)** Diagram of the genetic construction HisrMipartoxin-1. **(B)** Cleavage pET28a with XhoI and EcoRV on 1% agarose gel stained with ethidium bromide (Sigma, Saint Louis, MO, USA). MW: molecular weight marker (1 kb Plus DNA Ladder) (NEB); 1, 2, and 3: individual HisrMipartoxin-1 clones. The arrow indicates the 1578 bp expected fragment containing the 280 bp target insert.

The synthetic expression construct of Mipartoxin-1 (UniProt: B3EWF8) from UniProt [30] was obtained by optimization of rare codons in *E. coli* using the codon database Kazusa [31] and the OPTIMIZER tool [32]. To verify the open reading frame, the *in-silico* translation of the optimized sequence was performed by the ExPASy tool [33]. A N-terminal polyhistidine tag (6HisTag), a glycine-serine linker, and the TEV proteolytic site were added to the target sequence. Two restriction sites allowed cloning of the construct in the episomal plasmid pET28a: the NcoI site was included at the 5' end and the NotI site at the 3' end after the termination codon. The synthetic construct was cloned into the pUC57_BsaI_Free vector, and the plasmid product was named HisrMipartoxin-1 (Figure 1 A ). The construct synthesis was carried out by General Biosystems. 

### Cloning HisrMipartoxin-1

Chimiocompetent *E. coli* DH5α cells were transformed with the plasmid pUC57-rMipartoxin-1 or the empty pET28a vector by heat shock using the protocol described by Pope and Kent [34] with a modification in the heat-shocked time (40 sec). Transformed cells were recovered at 37 °C in Luria-Bertani broth (LB) for one hour and plated on LB plates that contained ampicillin (100 μg/mL) or kanamycin (50 μg/mL) as the selection antibiotic, respectively. Three individual colonies were grown overnight at 37 °C in 5 mL of LB medium supplemented with the respective antibiotic. The FavorPrep Plasmid Extraction Mini Kit (FAVORGEN Biotech Corporation, Wien, Austria) was used to isolate the plasmid DNAs contained in the *E. coli* strains, quantified with NanoDrop 2000 (Thermo Scientific, Waltham, MA, USA) and analyzed on 1% agarose gels stained with ethidium bromide. The plasmids and pET28a were cut with NcoI and NotI enzymes at 37 °C for three hours. The obtained fragments were gel-purified with the GeneJet Plasmid kit from Thermo Scientific (Waltham, MA, USA), ligated with T4 DNA ligase with a three-fold insert excess for two hours at room temperature, and finally transformed into chimiocompetent *E. coli* DH5α cells. Transformed bacteria were incubated at 37 °C for 15 hours in selection LB media with kanamycin. Propagation and recovery were as above. Transformation confirmation and directionality of the insert were done by XhoI and EcoRV cutting and agarose gel analysis. XhoI cuts after position 1573, and EcoRV cuts after position 158 on pET28a.

### Expression and purification of inclusion bodies 

BL21(DE3) *E. coli* cells were transformed with the recombinant plasmid pET28a-HisrMipartoxin-1 by heat shock (as above), and then one single colony was selected in LB-Kanamycin (50 mg/mL) and used for pre-culture at 37 °C overnight. HisrMipartoxin-1 expression and isolation from inclusion bodies were performed following the protocol described by Romero et al. [29]. In brief, 0.5 mM isopropyl-b-D-thiogalactopyranoside (IPTG) was added to the bacterial culture when it reached OD_600_ nm between 0.6 and 0.7 while growing at 37°C until eight hours after induction. Biomass was harvested by centrifugation, washed with phosphate-buffered saline (PBS) buffer (pH 7.4), and resuspended in lysis buffer 100 mM Tris- 10 mM EDTA at pH 8.5 for breaking the cells by sonication using an Ultrasonic Cell Disruptor (BIOBASE Biodustry, Shandong, China). The IBs were pelleted and dissolved at 16 h in equilibrium buffer (100 mM Tris- 10 mM EDTA- pH 8.0) in the presence of 8 M Urea and clarified by centrifugation. Refolding of HisrMipartoxin-1 was performed in dialysis tubing (SPECTRA / Por MWCO: 3.5 kDa) against a refolding buffer (200 mM Tris- 10 mM EDTA pH 8.5) with decreasing urea concentrations, starting 4 M up to 0.0625 M urea. Afterward, the recombinant solubilized protein was recovered by centrifuging at 32000 x g at 4 ºC for 30 min.

### Purification of HisrMipartoxin-1

The expressed HisrMipartoxin-1 protein was subjected to a two-step purification process: The first step consisted of a Ni-NTA (Ni-nitrilotriacetic acid) affinity chromatography using Ni-NTA agarose resin (Qiagen™ Ni-NTA Superflow) which was washed with the equilibrium buffer (this wash fraction was named ‘flow-through’). Non-specific proteins and cellular debris were removed using two washes (W1 and W2) with 20 mM imidazole and 500 mM NaCl while elution (E) of the recombinant protein was achieved with 360 mM imidazole buffer. To cut the 6His-Tag, TEV protease (10 mg/mL) was used in the presence of a dialysis buffer [20 mM Tris pH 8.5, 100 mM NaCl, 5 mM 2-Mercaptoethanol (Sigma, Saint Louis, MO, USA)] and a SPECTRA/Por MWCO: 3.5 kDa tubing membrane. This cleavage was performed to confirm the approximate molecular mass of rMipartoxin-1, which is the recombinant protein without the tag. The second purification step was performed using a Prominence-20A chromatograph (Shimadzu, Kyoto, Japan). For this, it was used reverse-phase high-performance liquid chromatography (RP-HPLC) on a C18 column (250 X 10 mm, 5 µm particle: Restek, Bellefonte, PA, USA). A linear gradient (0 to 70%) of an aqueous acetonitrile solution and 0.1% trifluoroacetic acid (TFA) at 1 mL/min for 35 min was applied. The elution signal was monitored at 215 nm with a photodiode detector (Shimadzu, Kyoto, Japan). Protein concentration (mg/mL) was determined using the Bradford protein assay [35].

### SDS-PAGE and western blotting 

Expression analysis of HisrMipartoxin-1 was assessed by SDS-PAGE in 14% Tris-tricine under reducing conditions according to Laemmli [36] and Schägger and von Jagow [37] protocols. Staining was made using Coomassie Brilliant Blue G-250 from Bio-Rad Laboratories (Hercules, CA, USA). A total of 10 µg of the samples and 3 µg of the molecular mass marker 11-250 kDa from New England Biolabs (Ipswich, MA, USA) were loaded. Immunodetection of the recombinant protein was carried out following the protocol of Lomonte [38] with some adaptations. Briefly, samples from SDS-PAGE were electrotransferred to nitrocellulose membranes (0.45 mm) for one hour in a TRANS-BLOT SD system (BIO-RAD, California, USA) using transference buffer (192 mM Glycine- 25 mM Tris-10% Methanol pH 8.3). A blocking solution (1% BSA/Casein) and a washing solution (1:10 of the 1% BSA/Casein blocking solution) were used. In addition, an anti-Mipartoxin-1 (1:100) coupled to peroxidase obtained from rabbits inoculated with Mipartoxin-1 was used. 

### Mass spectrometry 

The procedure for determining the molecular mass of the recombinant HisrMipartoxin-1 has been described in detail by Lomonte and Fernández [39] using a Q-Exactive Plus® Mass Spectrometer ESI-MS from Thermo Scientific (Waltham, MA, USA). Additionally, the monoisotopic masses were obtained by MS spectra deconvoluting with Freestyle® v.1.5 (Thermo Scientific, Waltham, MA, USA).

### Venoms and animals 

*M. mipartitus* venom (lyophilized) was obtained from adult specimens of both sexes collected by the Serpentarium of the University of Antioquia in the Antioquia region (Colombia). All *in vivo* experiments were performed using Swiss-Webster mice of both sexes of 18-20 g body weight. New Zeeland female rabbits (1.7 kg) were utilized for *in vivo* immunization experiments. All experiments were performed following protocols approved by the ethics committee of the University of Antioquia (License No. 110 of 2017).

### Lethal activity 

Lethality induced by HisrMipartoxin-1 was evaluated following the protocol described by Cecchini et al. [40]. A group of four mice each received several doses up to 100 µg/mouse (6 µg/g body weight) in 300 µL of salt solution (SS) by the intraperitoneal (i.p.) route, while a control group received SS alone. The number of dead mice 24 hours after the injection was recorded.

### Immunization and fractionation of anti-HisrMipartoxin-1 sera 

Immunization with HisrMipartoxin-1 was performed following the protocol described in detail by Lomonte [38] with some adaptations such as the first dose of 277 µg and boost doses 416, 624, 936, 1404, 1404, 2106, and 2106 µg of the recombinant HisrMipartoxin-1 protein. For all boosts, IFA was used. Four bleedings were performed thus: before being immunized (pre-immune or 0-day) and at 29, 70, 112, and 169 days after the first immunization. Rabbit immunoglobulins were obtained by the caprylic acid (Sigma, Saint Louis, MO, USA) method described by Steinbuch and Audran [41] from rabbit blood collected during the bleeding. The sera and IgG concentrations were quantified in a Nanodrop 2000 from Thermo Scientific (Waltham, MA, USA). Finally, sera were lyophilized and stored at -20 °C until used.

### Antibodies titers and immunological recognition 

The specific antibodies production against HisrMipartoxin-1 and immunological recognition of the specific rabbit sera were measured using an ELISA test following the protocol by Lomonte [38] with some modifications such as coating buffer (0.1 M Tris, 0.15 M NaCl pH 9.0) and washing buffer (PBS-0.05% Twin 20 pH 7.2). In short, 96 wells microplates (Falcon TM) were coated with 100 μL/well of 0.1 µg of venom *M. mipartitus* in coating buffer and incubated overnight at 4 °C. After, a blocking buffer (PBS-2% BSA) was added to wells for one hour and, subsequently, triplicate dilution curves of different sera and IgG were assayed separately (1:10, 1:100, 1:1000, 1:5000, and 1:10000). Two controls (serum pre-immune or non-immunized rabbit IgG was a negative control, and *M. mipartitus* venom was a positive control) were also included. After 1.5 h incubation, the plate was washed, and incubated with 100 µL of a dilution 1:8000 anti-rabbit IgG-peroxidase conjugate (Sigma, Saint Louis, MO, USA) for 1.5 h. After the last washing cycle was performed, the antibodies bound were observed by o-phenylenediamine (OPD), 2 mg/mL, and 30% H_2_O_2_. For absorbance readings, a Multiskan Sky Microplate Spectrophotometer from Thermo Scientific (Waltham, MA, USA) was used. The ELISA reaction was stopped by 0.32 M sulfuric acid. In another assay to evaluate cross-immunological recognition, HisrMipartoxin-1, Mipartoxin-1 [8], and Clarkitoxin-I-Mdum [9] were used to coat the plate, and the same procedure was followed as described.

### Lethal effect neutralization by anti-HisrMipartoxin-1-IgG 

To evaluate the neutralization ability of the immunoglobulins purified from anti-HisrMipartoxin-1 serum, several doses of anti-HisrMipartoxin-1-IgG were mixed with 1.5 LD50 (13.5 µg/mouse) [42] of *M. mipartitus* venom or 1.5 LD50 (9 µg/mouse) of Mipartoxin-1 [43], both in 300 µL of SS. These preparations were incubated for a half-hour at 37 °C and then injected by intraperitoneal (i.p.) route to three mice. The group that received the whole venom was used as a control, and observations were continued until 24 h after injection [44].

### Statistical analysis 

Results were expressed as the mean ± SD; a one-way ANOVA with Bonferroni post-test was used to determine significant differences (p < 0.05).

## Results

### HisrMipartoxin-1 cloning 

The coding sequence of Mipartoxin-1 was optimized to *E. coli*-favored codons. The construction incorporated the cleavage sites NcoI, NotI, and XbaI. The first two were included for the directional insertion into pET28a, while XbaI was added for future study. To conserve the open reading frame, a cytosine before the 6His-Tag was included, in addition to a proline and an alanine residue prior to the glycine-serine (GSGSGS) linker to confer flexibility to the histidine tail. Seven amino acids (ENLYFQG), corresponding to the cleavage site of the protease TEV, were included before the toxin sequence, and a UAG was included downstream of the target sequence. Cleavage of plasmid DNA and pET28a with NcoI and NotI endonucleases resulted in two fragments: one of the expected size (280 bp) and another of 5239 bp corresponding to pET28a (Figure 1 A , B). The growth of transformed *E. coli* DH5α colonies with the ligation product between the insert and pET28a was evidence of successful cloning. Transformed clones were confirmed by restriction analysis with XhoI and EcoRV yielding a fragment of 1578 bp and another fragment of 3958 bp (Figure 1 B ). The first fragment matched the insert (280 bp) expected size, plus a 1411 bp fragment (EcoRV-XhoI) without 113 bp released in cut, and the second fragment matched with a fragment spanning the XhoI-EcoRV segment.

### Expression, purification, and lethal activity of HisrMipartoxin-1 

**Figure 2.**
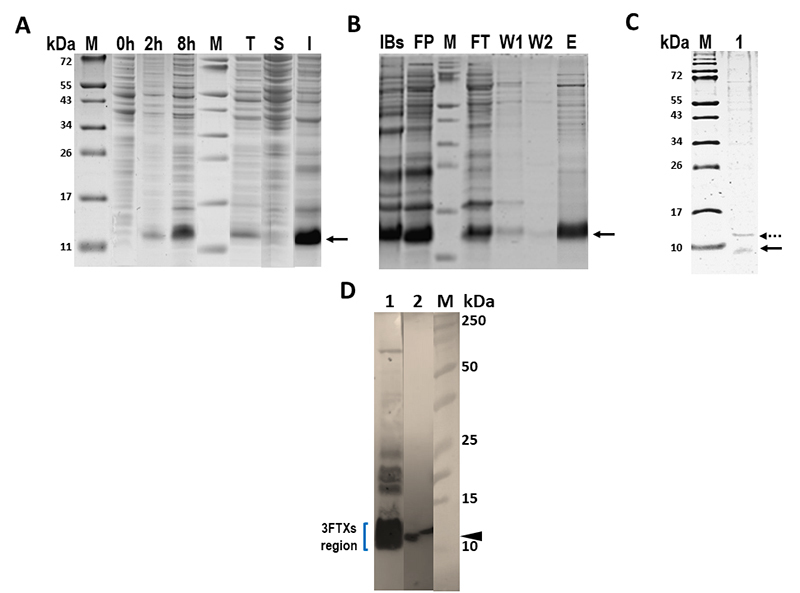
HisrMipartoxin-1 expressed in *E. coli* BL21 (DE3) strain. **(A)** SDS-PAGE analysis of HisrMipartoxin-1 before (at 0 hours) and after IPTG induction (at 2 and 8 hours). M: molecular mass marker; T: total protein; S: soluble fraction; I: insoluble fraction. **(B)** *In vitro* refolding and isolation of HisrMipartoxin-1. IBs: solubilized inclusion bodies; RP: refolded protein; FT: Flow-through; W1 and W2: Wash 1 and 2; E: Elution. **(C)** Cleavage of 6His-tag with TEV protease. rMipartoxin-1 was detected as a band of about 8 kDa (black arrow). HisrMipartoxin-1 showed approximately 11 kDa (dashed arrow). **(D)** Western blot analysis using the anti-Mipartoxin-1 antibody. Lane 1: The *M. mipartitus* venom profile contained different proteins; the region around 10 kDa is enriched in 3FTxs, particularly Mipartoxin-1 (7 kDa). The intensity of the band is due to their abundance in the *M. mipartitus* proteome. Lane 2: The molecular mass of HisrMipartoxin-1 was approximately 11 kDa (arrow). M: molecular mass marker in kDa.

**Figure 3.**
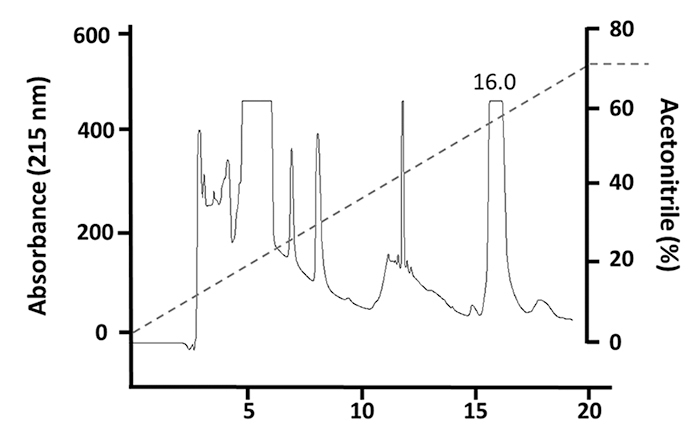
Purification by RP-HPLC chromatography. HisrMipartoxin-1 eluted at 16.0 min. An acetonitrile linear gradient and 1 mL/min flow were applied for elution.

The HisrMipartoxin-1 recombinant protein was expressed in *E. coli* strain BL21 (DE3) as a histidine hexamer-tagged (His-tag) fusion protein. HisrMipartoxin-1 was detected by SDS-PAGE (Figure 2 A ) as an insoluble protein (I) in inclusion bodies. HisrMipartoxin-1 was subjected to *in vitro* folding and then recovered after two purification steps using agarose nickel affinity chromatography and further purification by RP-HPLC. After purification, the total yield of HisrMipartoxin-1 was 13.5 mg per liter of culture medium. The molecular mass of HisrMipartoxin-1 under reduced conditions after β-mercaptoethanol treatment was approximately 11 kDa (Figure 2 A , arrow), close to the theoretical molecular mass (9887 Da). Furthermore, the molecular mass of HisrMipartoxin-1 determined by mass spectrometry was 9841 Da. As shown in Figure 2 B , a gradual increase of expression of HisrMipartoxin-1 was evident as a band with a molecular mass of about 11 kDa, detected after two to eight hours of IPTG induction. The bacterial growth time to a density of 0.6-0.7 before induction with IPTG was considered as the initial time of 0 hours. The total-cell protein (T) content showed -besides HisrMipartoxin-1- several proteins of different molecular sizes with 0.5 mM IPTG induction. The insoluble (I) material resulting from cell lysate consisted of a thick band, and the soluble (S) fraction presented an electrophoretic profile different from the insoluble fraction, that is, no band was evident in the molecular mass detected in the insoluble fraction. Figure 2B shows the solubilized inclusion bodies (IBs) and the refolded HisrMipartoxin-1 protein (RP). The refolded HisrMipartoxin-1 was purified using nickel affinity chromatography. In all four fractions (flow-through, wash 1, wash 2, and elution), the presence of a band was noted of the expected molecular mass of HisrMipartoxin-1 (Figure 2 B , arrow). The molecular mass of rMipartoxin-1 was confirmed after cutting the histidine tail. As shown in Figure 2 C , a partial cut of the histidine tail was evident as two bands: a predominant band of the expected rMipartoxin-1 size (about 8 kDa), which was close to the reported molecular mass (~7 kDa) [8] and another band of about ~11 kDa corresponding to tagged HisrMipartoxin-1 protein. HisrMipartoxin-1 was also detected by western-blot assay (Figure 2 D ). Given that the tag cleavage was incomplete and the amounts of the recombinant protein that can be lost during the purification process decreased the total yield, we decided to continue using HisrMipartoxin-1 in all essays. Then, as a result of RP-HPLC purification, HisrMipartoxin-1 was identified at an elution time of about 16.0 min (Figure 3). To confirm the lethality of the purified toxin, both rMipartoxin-1 and HisrMipartoxin-1 were tested in mice. Neither caused lethality in mice of 18-20 g of body weight (n = 4) in doses up to 100 µg/mouse. 

### Evaluation of anti-HisrMipartoxin-1 titers in serum 

**Figure 4.**
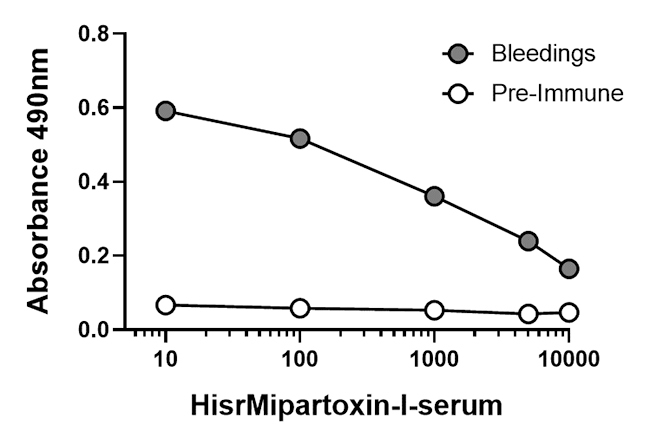
Antibody titers by ELISA in serum (average of four bleeding) against HisrMipartoxin-1. Sera from four bleedings in different dilutions (1:10 to 1:10000) were used, and absorbances were recorded at 490 nm. * Indicates differences with the pre-immune serum (p < 0.0001). Data correspond to the mean ± SD (n = 3).

One rabbit was immunized with HisrMipartoxin-1 for six months, using a scheme that started with 277 µg of recombinant immunogen with an increase from 1.5 until the fifth immunization, one sustained dose in the sixth, followed by another increase from 1.5, and one final sustained dose in the eighth immunization. The serum samples of four bleedings were evaluated for their antibody levels against HisrMipartoxin-1. Results showed an increase of antibody titers against HisrMipartoxin-1 until 1:10000 (Figure 4). Differences were detected compared with the pre-immune serum (p < 0.0001). 

### IgG purification, titers, and immunological cross-recognition 

**Figure 5.**
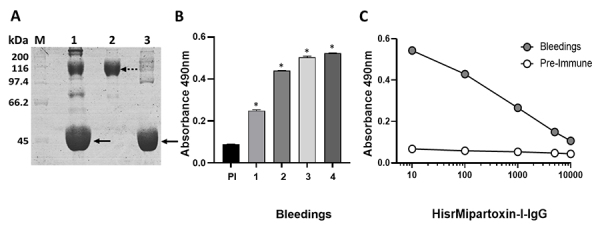
IgG purification, immunorecognition, and anti-HisrMipartoxin-1-IgG titers**. (A)** Fractionation IgG of hyperimmune serum using caprylic acid. Efficiency analysis was made by 10% SDS-PAGE under non-reducing conditions. The gels-stained with Coomassie Blue R-250. M: broad range molecular marker (kDa). 1: anti-HisrMipartoxin-1 serum; 2: IgG obtained after fractionation; 3: Albumin standard. The dashed arrow indicates the IgG band, and the black arrow indicates albumin. **(B)** Immunorecognition by ELISA of anti-HisrMipartoxin-1-IgG from each bleed (bleedings 1 to 4), and pre-immune serum (PI) against HisrMipartoxin-1. A 96-well plate was coated with HisrMipartoxin-1, and IgG from each bleeding was used at a 1:100 dilution (from an initial concentration of 50 mg/mL). The pre-immune serum was used at the same dilution. **(C)** Titration curve of anti-HisrMipartoxin-1-IgG by ELISA against HisrMipartoxin-1. IgG from four bleedings was used in dilutions from 1:10 to 1:10000 (from an initial concentration of 50 mg/mL). For B and C, * indicates differences compared to the pre-immune serum (p < 0.0001). Data correspond to the mean ± SD (n = 3).

**Figure 6.**
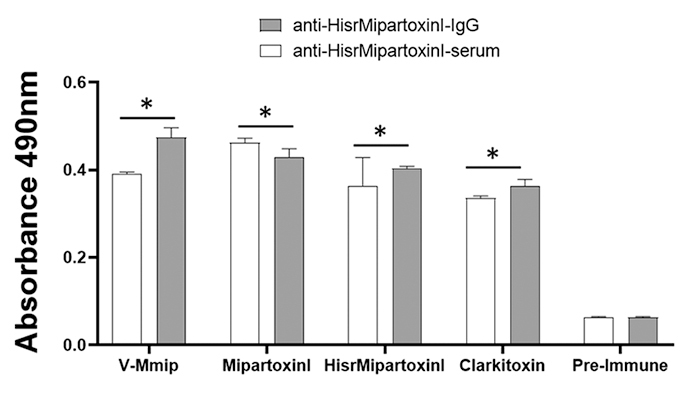
Immunoreactivity by ELISA of sera and IgG anti-HisrMipartoxin-1 against *M. mipartitus* venom (V-Mmip), Mipartoxin-1 (native), HisrMipartoxin-1, and Clarkitoxin-I-Mdum. *Indicates differences when compared to the pre-immune serum (p < 0.0001). Data correspond to the mean ± SD (n = 3).

The fractionation of sera with caprylic acid allowed for obtaining IgG and reduction of albumin (Figure 5 A ). The anti-HisrMipartoxin-1-IgG yield was about 11.8 mg/mL. IgG was prepared at a concentration of ~50 mg/mL. Results showed an increase in IgG titers against HisrMipartoxin-1 from the first immunization (Figure 5 B ) and up to dilutions of 1:10000 (Figure 5 C ). Also, differences were detected in comparison to pre-immune IgG (p < 0.0001). Antibodies detected in serum and anti-HisrMipartoxin-1-IgG isolated showed reactivity against its recombinant immunogen, and cross-reactions with Mipartoxin-1, *M. mipartitus* venom, and Clarkitoxin-I-Mdum were detected by ELISA (Figure 6). 

### Neutralization of the lethal effect of Mipartoxin-1 by anti-HisrMipartoxin-1-IgG 

Anti-HisrMipartoxin-1-IgG neutralized 100% of the lethal effect of Mipartoxin-1, using a dose of 2 mg/mouse against 1.5 DL_50_ of native toxin (9 µg). Lower doses did not inhibit the lethal effect. Additionally, anti-HisrMipartoxin-1-IgG did not neutralize the lethal effect of the *M. mipartitus* whole venom (n = 3) (results not shown)*.*

## Discussion 

Owing to the scarcity of *Micrurus* venoms, investigations have focused on the biochemical and pharmacological properties of whole venoms and the properties of isolated toxins. However, in recent years, biotechnological developments and improved chromatographic methods have greatly simplified the expression and purification of recombinant toxins from some species of *Micrurus* intending to improve antivenom production and solve, in part, the venom scarcity.

In general, snake venoms are a cocktail of pharmacologically active proteins and peptide components that can be enzymatic or nonenzymatic [45]. Most of these components are immunogenic (~98% of their dry weight), including toxins that induce both severe and non-toxic effects [46]. One of the most important toxin families in elapid venoms are the 3FTxs, neurotoxins whose primary target is the peripheral nervous system and cause neuromuscular weakness and paralysis [47]. The most abundant 3FTx (Mipartoxin-1) of *M. mipartitus* venom was isolated and sequenced by Rey-Suarez et al. [8]. Mipartoxin-1 is lethal and causes neurotoxic effects [8]. Cardona-Ruda et al. [43] recently demonstrated that antibodies produced against Mipartoxin-1 (Mm8) and MmipPLA_2_ (Mm20) neutralize *M. mipartitus* whole venom. This work presents the production of recombinant Mipartoxin-1 and its immunogenic potential.

Mipartoxin-1 was produced in an expression plasmid encoding a synthetic gene codon optimized for *E. coli*. The construct HisrMipartoxin-1 overexpressed the target protein in *E. coli* BL21 (DE3), and its yield was 13.5 mg/L after purification. Protein yields in the range of 10-50 mg/L correspond to medium-level expression for recombinant proteins, as has been reported [48, 49]. It is important to stress that *E. coli* strains differentially express heterologous genes, and intrinsic features of the pET vector can also be a determining factor for achieving different expression levels of the recombinant proteins [50]. The yield (13.5 mg/L) of HisrMipartoxin-1 after purification is comparable with yields obtained for a different type of snake venom protein such as PLA_2_s: for instance, Romero-Giraldo et al. [29] obtained a yield of 10.1 mg/L for His-rMdumPLA_2_, a PLA_2_ from *M. dumerilii* expressed in the BL21 (DE3) strain from *E. coli* using pET28a as the expression vector. 

Several authors have reported yields for other recombinant 3FTXs from different species [22-23, 51-53] and other low molecular weight toxins such as PLA_2_s [54], which vary according to the *E. coli* strain, the plasmid vector, and expression conditions. In this context, it has been posed that recombinant protein yields may be associated with several factors, such as the growth rate of the host cells and the absolute amount of soluble protein produced per culture volume [55], the His-tag that confers stability to the protein [56], the vector backbone and design and the cloning strategy [29, 57]. 

The mass of HisrMipartoxin-1 detected by SDS-PAGE is close to its theoretical molecular mass (9887 Da), however, it differed from the native Mipartoxin-1, which has a molecular mass of 7030 Da [8]. The difference between the deduced molecular mass and the SDS-PAGE mobility for HisrMipartoxin-1 is a consequence of the elements included in the genetic construct, such as the histidine segment, the linker GS, and the TEV protease sequence. The rMipartoxin-1 molecular mass after the tag cleavage was comparable to the native toxin (Mipartoxin-1), evidenced in the TEV protease cleavage assay.

HisrMipartoxin-1 was expressed as IBs, hence it had to be solubilized with a denaturing agent, such as urea. However, in contrast to the native protein, solubilization of the HisrMipartoxin-1 IBs did not result in a protein with biological activity [8]. This result is due to non-native intermolecular and intramolecular interactions during the refolding process [58], which may produce misfolding or incomplete protein folding [59, 60] with the formation of non-native disulfide bonds [61], which together generate a non-native protein. It should be noted that the cysteine pattern is a highly conserved feature among 3FTXs [62], just like Tyr25 and Phe27 residues, which play a key role in correct folding [63]. Therefore, any change in the primary structure may affect biological functions and the molecular targets [64]. On the other hand, considering that the activity of the protein is closely correlated to its native structure [65, 66], another possible explanation is that although the presence of the His-tag confers stability to recombinant protein [56], also may cause negative effects on the tertiary structure during the refolding process, altering target and/or inhibitor interaction sites or the biological activity of the protein [67], a subject that warrants further study. Nonetheless, it is documented that the insertion of sequences in protein structures may decrease conformational stability and cause the loss or reduction of biological activity [68]. In addition, impurities that result during the solubilization process of IBs can interact with the expressed protein and interfere with its proper folding [58].

Moreover, it is important to note that the percent active protein recovered during *in vitro* refolding is less than 50% [69], and in other cases, no biologically active protein is obtained [70]. Taken together, HisrMipartoxin-1 adopted a stable soluble conformation, but its crucial fold -typical of the 3FTxs- consisting of three β-sheet loops with all four conserved disulfide bridges [62] may not have been obtained, therefore lethal activity was not observed. In fact, Girish and colleagues [71] have suggested that the biological activities of 3FTxs might be associated with slight conformational differences in the three β-sheet loops, inferring that a small change in the protein structure could alter its functionality. However, confirmation of this hypothesis needs further studies. 

Western-blot analysis showed that the native Mipartoxin-1 antiserum recognized HisrMipartoxin-1. Similarly, this antiserum recognized the 3FTxs region present in *M. mipartitus* venom. Additionally, rabbit polyclonal antibodies generated against HisrMipartoxin-1 recognized *M. mipartitus* venom, native Mipartoxin-1, and Clarkitoxin-I-Mdum from *M. dumerilii*. It is possible that some antigenic determinants were recognized by the anti-HisrMipartoxin-1 antibodies, which were generated because of the rabbit immune response. In addition, considering that *Micrurus* antivenoms have variable immunological cross-reactivity according to different authors [72-76] and a cysteine pattern of the short-chain 3FTxs (or “type-I”) [77] conserved in many elapid venoms [9], the immunological cross recognition of antibodies against HisrMipartoxin-1 might be a consequence of a conserved backbone of both native toxins. While it is true that the Clarkitoxin-I-Mdum and Mipartoxin-1 sequences share only 26% identity between amino acid sequences [9], this study shows that the possible structural changes of the recombinant protein favored some conserved antigenic determinants resulting in the cross-recognition against 3FTx other than Mipartoxin-1. In addition, this result contrasts with the study from Rey-Suárez et al. [9], who reported that the anti-Mipartoxin-I or anti-Clarkitoxin-I-Mdum sera recognized components in *M. mipartitus* and *M. clarki* venoms, but did not cross-recognize their homologous antigen, Mipartoxin-1, and Clarkitoxin-I-Mdum, respectively.

Our results showed that the immune response was evident by the presence of antibodies against HisrMipartoxin-1 from the first immunization, indicating that the recombinant immunogen generated an immunological response. Although antibody titers were low, it is known that 3FTxs, being low molecular mass proteins, generate a low immune response [38, 78-79]. Proof of this is the results obtained by Laustsen et al. [80], Fernández et al. [81], and Cardona-Ruda et al. [43], who showed that 3FTxs induced a lower immune response compared to the PLA_2_s. Nevertheless, these groups of toxins are responsible for the most important toxic effects observed in coral snakebite victims [5, 14]. Further, HisrMipartoxin-1 did not induce toxic effects, which could be an advantage since it reduces the possibility of inducing toxicity during the immunization process.

Additionally, it was found that anti-HisrMipartoxin-1-IgG completely neutralized the lethal effect of the native Mipartoxin-1. Toxin neutralization has been considered to take place when the antibody is bound by the variable region [80]. It is possible to hypothesize that some epitopes are located on conserved structures, specifically on loop II of the 3FTx, as has been reported [22, 82]. However, this hypothesis needs to be proved in further studies. It has also been reported that recombinant proteins from IBs produce neutralizing antibodies [83]. In contrast, although the antibodies did not neutralize the *M. mipartitus* whole venom, these were highly recognized in the whole venom. This is because another lethal component has been reported named MmipPLA_2_ (LD_50_ 0.85 µg/mouse [7]), the most abundant PLA_2_ from *M. mipartitus* venom. Also, as mentioned above, similar findings were reported by Cardona-Ruda et al. [43], who used IgG against the native toxin and demonstrated that the MmipPLA_2_ toxin was involved in the lethal effect and that the lethal effect of the venom was neutralized only with a mixture of antibodies against these two toxins (Mipartoxin-1 and MmipPLA_2_) [7, 43]. Given that HisrMipartoxin-1 generated non-neutralizing antibodies against venom *M. mipartitus*, uncover the need to include other key toxins of *M. mipartitus*, in the production of anti-*Micrurus* antivenoms to improve the neutralizing ability against this species.

Consistent with the findings above and considering that neutralization of single toxins by antibodies may notably reduce the clinical manifestations of envenoming given their high toxicity and high abundance in the venom [80, 84], the development of recombinant proteins containing epitopes from the main toxic components from *M. mipartitus* venom could be a powerful source of key antigens for the preparation of neutralizing antibodies [85]. Likewise, it is important to highlight that immunization with a snake venoms mixture [86, 87] or a mixture of their main toxins [88] increases the neutralization scope of the antivenom. In this sense, mixtures of antibodies against key toxins can result in an efficient strategy to neutralize most of the medically relevant snake venom toxins [27, 84, 89-92]. 

Ultimately, this work demonstrates that HisrMipartoxin-1 induces antibodies in an animal model with the neutralization ability of Mipartoxin-1, one of the toxins responsible for the lethal effect of *M. mipartitus* venom. Thus, this recombinant toxin can be used as an immunogen to improve the development of an anticoral antivenom and overcome the limitations associated with the availability of *M. mipartitus* venom.

## Conclusion

In this study, the heterologous expression of HisrMipartoxin-1, the most abundant 3FTx of *M. mipartitus* venom (Mipartoxin-1), was achieved using E*. coli* BL21 (DE3). This work is the first report of a recombinant 3FTx from *M. mipartitus* venom. HisrMipartoxin-1 induced antibodies that neutralized the lethal effect of the native toxin. These results advance the development of anticoral antivenom using recombinant toxins and offer a solution to the limited access and limitations in the availability of venom from *Micrurus* species.

### Abbreviations

BP: base pairs; BSA: Bovine serum albumin; EDTA: Ethylenediaminetetraacetic acid; ELISA: Enzyme-Linked Immunosorbent Assay; GS: Glycine Serine; IB: Inclusion body; IFA: Incomplete Freund's adjuvant; INS: Instituto Nacional de Salud; i.p.: Intraperitoneal route; IPTG: isopropyl-b-D-thiogalactopyranoside; kDa: Kilodalton; LD: Lethal dose; LB: Luria-Bertani broth; MWCO: molecular-weight cutoffs; Ni-NTA: Ni-nitrilotriacetic acid; nm: nanometer; OPD: o-phenylenediamine; PBS: phosphate-buffered saline; PLA_2_: Phospholipase A_2;_ RP-HPLC: Reversed Phase-High-performance liquid chromatography; SD: Standard deviation; SDS-PAGE: Sodium dodecyl-sulfate polyacrylamide gel electrophoresis; SS: Salt solution; TFA: Trifluoroacetic acid; WHO: World Health Organization; 3FTx: three finger toxins.
